# Oxidant-Sensing Pathways in the Responses of Fungal Pathogens to Chemical Stress Signals

**DOI:** 10.3389/fmicb.2019.00567

**Published:** 2019-03-19

**Authors:** Hiba Simaan, Sophie Lev, Benjamin A. Horwitz

**Affiliations:** ^1^Faculty of Biology, Technion – Israel Institute of Technology, Haifa, Israel; ^2^Centre for Infectious Diseases and Microbiology, The Westmead Institute for Medical Research, Westmead, NSW, Australia

**Keywords:** signaling, transcription factor, Yap1, Hog1, xenobiotics

## Abstract

Host defenses expose fungal pathogens to oxidants and antimicrobial chemicals. The fungal cell employs conserved eukaryotic signaling pathways and dedicated transcription factors to program its response to these stresses. The oxidant-sensitive transcription factor of yeast, YAP1, and its orthologs in filamentous fungi, are central to tolerance to oxidative stress. The C-terminal domain of YAP1 contains cysteine residues that, under oxidizing conditions, form an intramolecular disulfide bridge locking the molecule in a conformation where the nuclear export sequence is masked. YAP1 accumulates in the nucleus, promoting transcription of genes that provide the cell with the ability to counteract oxidative stress. Chemicals including xenobiotics and plant signals can also promote YAP1 nuclearization in yeast and filamentous fungi. This could happen via direct or indirect oxidative stress, or by a different biochemical pathway. Plant phenolics are known antioxidants, yet they have been shown to elicit cellular responses that would usually be triggered to counter oxidant stress. Here we will discuss the evidence that YAP1 and MAPK pathways respond to phenolic compounds. Following this and other examples, we explore here how oxidative-stress sensing networks of fungi might have evolved to detect chemical stressors. Furthermore, we draw functional parallels between fungal YAP1 and mammalian Keap1-Nrf2 signaling systems.

## Introduction

Fungi have extraordinary capacity to adapt to a changing environment, responding to environmental cues via signaling pathways poised for activation by specific stimuli. As microbes, fungal cells are exposed to a multitude of stimuli, many of which are interpreted as stressors (Brown et al., 2017; Rangel et al., 2018). These stimuli include xenobiotics or reactive oxygen species of extra- or intra-cellular origin. Signal transduction is mediated by transcription factors that reprogram gene expression to cope with the stress. Transcriptional responses to different stresses often overlap, and there is evidence that xenobiotic and oxidative stress response mechanisms are evolutionarily related. Although yeast commonly serve as a model to help understand general cell biological principles, with respect to the question posed here, the mammalian Keap1-Nrf2 pathway has also been extensively studied due to its central importance in stress response at the cellular level, and, more broadly, human disease. This pathway is activated by both xenobiotics and reactive oxygen species and mediates detoxification of both. In a review aptly titled “an evolutionary journey through stressful space and time” (Fuse and Kobayashi, 2017) chart the evolution of the Keap1-Nrf2 signaling cascade. Of interest to us here is the parallel made between yeast YAP1 sensing and the KEAP-NRF2 pathway, although the biochemical details are quite different. The functional link between the response to xenobiotics and oxidants is conserved, and relies on modification of thiol groups in signaling molecules, triggering conformational changes, and altering protein–protein interactions. This suggests that there may be a fundamental need for this linkage, much as diverse organisms like *Neurospora*, flies and mammals have similar circadian clock mechanisms, although the sequences of the central transcription factors share little or no homology. Here we will explore how xenobiotic and oxidant stress responses are linked in fungi. The emphasis is on how this network appears to have evolved in filamentous pathogens. We discuss how research continues to be guided by the yeast model, with unique properties in the filamentous species that might be targeted for basic and applied approaches.

## Yap1, a Sensor of Oxidants Which Also Responds to Chemicals

### YAP1, a Dedicated Redox-Sensitive Fungal Transcription Factor

Yeast YAP1 is a bZIP transcription factor, which binds as a dimer to the YAP1 response element (YRE) TTA (C/G) TAA. Yap1 contains N-terminal (n-CRD, located near the center of the molecule, actually) and C-terminal (c-CRD) cysteine-rich motifs which can form multiple intramolecular disulfide bonds, masking the nuclear export signal (NES) located within the C-terminal cysteine-rich motif. When exposed, NES of YAP1 is recognized by the exportin Cmr1 to remove YAP1 from the nucleus. Activation of YAP1 by hydrogen peroxide has been studied in detail and involves formation of 1–3 disulfide bonds between six cysteines of YAP1 in a multi-step process mediated by thiol peroxidase Orp1/Gpx3, rendering YAP1 reduction-resistant (Okazaki et al., 2007). Yap1 is found in a complex with Yap1-binding protein (Ybp1), essential for its activation by H_2_O_2_. First, Cys36 of Gpx3 senses the H_2_O_2_ signal and is oxidized to sulfenic acid Cys36-SOH. Second, Ybp1 brings together Gpx1 and Yap1 to form ternary complex in which Gpx3 forms an intermolecular disulfide bond Cys36-Cys598 with Yap1. This bond is subsequently converted to the intramolecular Yap1 Cys303-Cys598 bond. Gpx3 Cys36 forms an intramolecular bond with Gpx3 Cys82. In the process of H_2_O_2_-mediated Yap1 activation Ybp1 operates as a signaling scaffold, or a “sulfenic acid chaperone” selectively promoting disulfide bond formation between Gpx3 and Yap1 (Delaunay et al., 2002; Veal et al., 2003; Toledano et al., 2004; Bersweiler et al., 2017). In a third step, an additional bond between n-CRD and c-CRD of Yap1 is created (Cys310-Cys629). Lastly, all six cysteines are thought to become engaged in n-CRD – cCRD intramolecular association (Okazaki et al., 2007). Reduced Orp1/Gpx3 as well as reduced YAP1 are eventually regenerated by reaction with reduced thioredoxin (Delaunay et al., 2002; Wood et al., 2003, 2004). As a result of oxidation, YAP1 accumulates in the nucleus and triggers induction of antioxidant genes, including GSH1 (γ-glutamylcysteine synthetase), GPX2 (glutathione peroxidase), TRX2 (thioredoxin), TSA1 (thioredoxin peroxidase) and others.

### YAP1 Is Activated by Diverse Stimuli Which May or May Not Be Accompanied by Oxidative Stress

In addition to ROS, YAP1 mediates resistance to heavy metals and toxic xenobiotics. The mechanism of YAP1 activation depends on the activating stimulus: Gpx3 mediates formation of intramolecular cysteine bonds in response to H_2_O_2_, as described above, while thiol-reactive xenobiotics were shown to activate YAP1 directly, by binding to its cysteines (Azevedo et al., 2003). This mechanism of YAP1 activation does not depend on the primary oxidative stress sensor, Gpr3, or Ybp1. Toxic metals including cadmium, arsenic and cobalt potentially engage both mechanisms of YAP1 activation due to their propensity to trigger general increase in ROS (Takeuchi et al., 1997; Azevedo et al., 2003; Menezes et al., 2008; Pimentel et al., 2014; Caetano et al., 2015). This twofold manner of YAP1 activation was also described for a quinone, menadione, which is both electrophile and a superoxide anion generator (Azevedo et al., 2003). Electrophile N-ethylmaleimide and a by-product of glycolysis, methylglyoxal, also activate YAP1 independently of oxidative stress, via formation of direct adducts to the c-CRD cysteines (Cys598, Cys620, and Cys629) of YAP1 (Azevedo et al., 2003; Maeta et al., 2004; Takatsume et al., 2006). Likewise, there is evidence that allicin, a thiol-reactive sulfur-containing natural product from garlic with broad antimicrobial effects, activates YAP1 by direct modification of the c-CRD cysteines (Gruhlke et al., 2017). Similarly, In *Candida albicans*, CAP1 has been shown to have a role in resistance to hydrogen peroxide, cadmium, iron chelator 1,10-phenanthroline and radical-producing drug, 4-nitroquinoline *N*-oxide (Alarco and Raymond, 1999). Thus, flexibility of the Yap1 activation mechanism potentially allows it to be activated by any thiol-reactive foreign compound, including drugs, industrial pollutants and other xenobiotics. While nuclear targeting of YAP1 in response to hydrogen peroxide triggers marked up-regulation of antioxidant genes, YAP1-dependent detoxification of xenobiotics relies on different genes, such as vacuolar efflux pump YCF1 that belongs to the ATP-binding cassette (ABC) transporters and major facilitator (MFS) superfamily transporters FLR1 and ATR1 (Wemmie et al., 1994; Alarco et al., 1997). Deletion of ATR1 or FLR1 results in increased sensitivity to the phenolic phenylpropanoid, coniferyl aldehyde, in *Saccharomyces cerevisiae* (Sundström et al., 2010).

**FIGURE 1:**
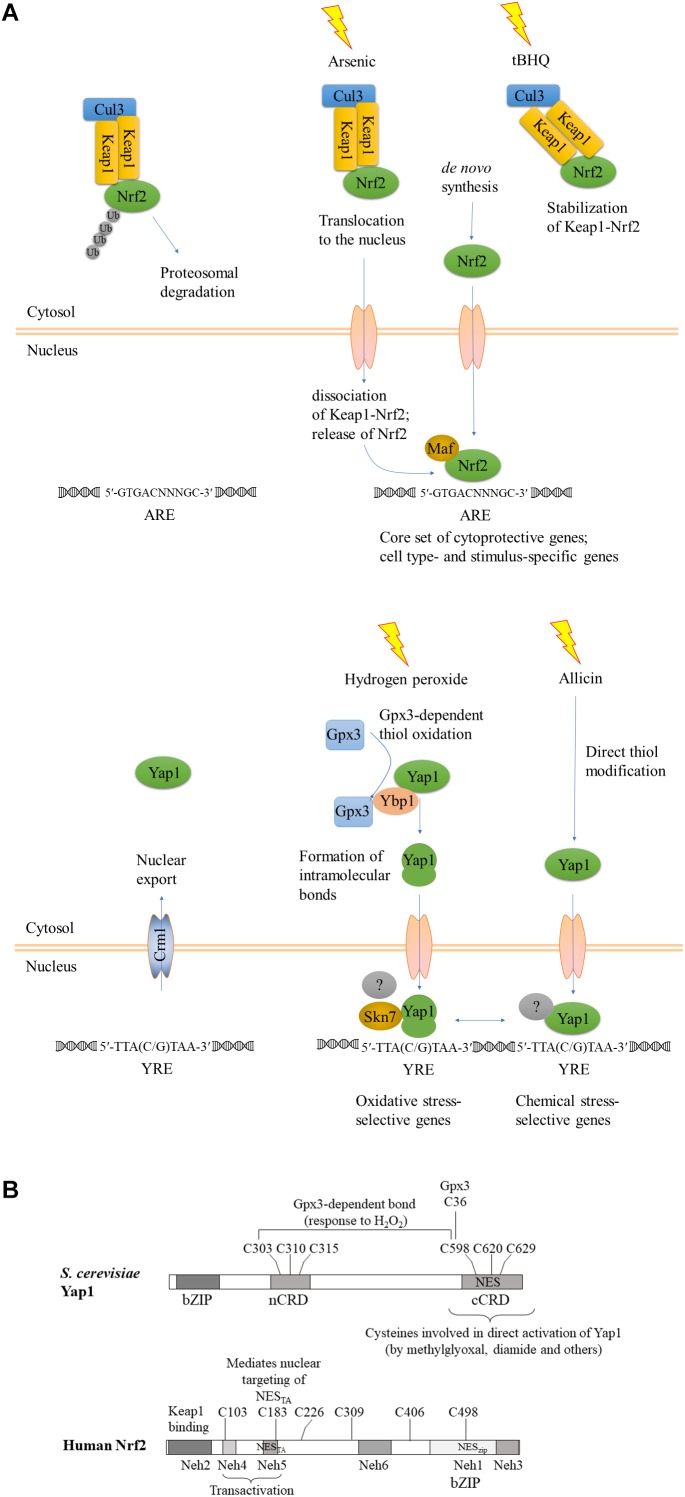
Evolutionary conservation of functional reaction to oxidant and chemical stress signals. **(A)** A schematic view, after (Fuse and Kobayashi, 2017), modified to emphasize the convergent functions and comparison between Keap1-Nrf2 and YAP1. KEAP1, Kelch-like ECH-associated protein; Nrf2, Nuclear factor-erythroid 2 p45-related factor 2; Maf, small musculoaponeurotic fibrosarcoma protein; YAP1, yeast AP-1. Upper panel, Keap1-Nrf2; lower panel, yeast YAP1. The gray-shaded transcription factors with a question mark indicate different transcriptional regulatory complexes at different target genes. **(B)** Structural maps of *S. cerevisiae* YAP1 and human Nrf2 with all cysteines indicated. Domains of Nrf2 were mapped according to (Fuse and Kobayashi, 2017).

Interestingly, YAP1 has different modes of activation and can regulate expression of different targets, depending on the type of stress. At the structural level, there are two known modes of activation: intramolecular disulfide bridge formation upon exposure to oxidants, and direct reactivity of the c-CRD cysteines to other electrophilic chemicals and heavy metal cations (Azevedo et al., 2003). The question of whether there are also distinct downstream targets was later addressed genome-wide: a transcriptomic comparison of wild type and isogenic delta-YAP1 yeast strains showed that H_2_O_2_ and an electrophile, the thiol-reactive Michael acceptor N-ethylmaleimide (NEM) induced the expression of distinct sets of genes. Indeed, study of the adaptive responses to H_2_O_2_ and NEM showed that they did not cross-protect. At a 1.5-fold cutoff for the up-regulated genes, this analysis revealed 43 genes specifically responding to H_2_O_2_, 239 genes responding to both NEM and acrolein, and 214 genes responding to all three chemicals (Ouyang et al., 2011). How distinct sets of genes are regulated by the same transcription factor following nuclear retention is not yet clear. Study of co-activators and co-repressors is helpful. Indeed, YAP1 joins distinct transcriptional complexes depending on the target gene. The transcriptional Mediator complex interacts differently depending on the target gene and the specific oxidant. Mediator head module subunits Med18, Med20, and Med19 join the transcriptional complex at the *CYC1* promoter, which is poised by preloaded polymerase RNAPII and activated by YAP1 in response to oxidative stress. Furthermore, the oxidant-activated, disulfide-bridged form of YAP1 is required not only for nuclear retention, but also for transcriptional activation of the *TRX2* promoter. The activated, folded form of YAP1 is required to recruit the mediator subunit ROX3 (Gulshan et al., 2005; Lee et al., 2013). Another important partner of YAP1 at oxidative stress response promoters is the two-component response regulator regulator Skn7 (Lee et al., 1999; Mulford and Fassler, 2011); for a schematic view see Figure 1, 4.

### Filamentous Fungal Orthologs of YAP1: fAP1’s

*Saccharomyces cerevisiae* YAP1 orthologs were identified in other yeasts and filamentous fungi, where they are also involved in response to oxidative and other stresses. The cysteine residues in the C-terminal cysteine-rich domain are almost perfectly conserved from yeast through the Dikarya. However, there is only one cysteine residue in the n-CRD that is conserved between yeast species and filamentous fungi (Cartwright and Scott, 2013). Here, we will abbreviate filamentous fungal orthologs of YAP1 as fAP1s, for example: YAP1 is yeast (*S. cerevisiae*) AP1; PAP1 is *Saccharomyces pombe* AP1; ChAP1 is *Cochliobolus heterostrophus* AP1). fAP1s are needed for resistance to oxidative stress. In several pathogens that have been studied, including *Ustilago maydis*, *Magnaporthe oryzae*, and *Alternaria alternata*, fAP1 is a major factor responsible for oxidative stress resistance and virulence on the host (Molina and Kahmann, 2007; Lin et al., 2009, 2011; Guo et al., 2011). The orthologs of YAP1 in *A. alternata*, the rice blast fungus *M. oryzae* and *Colletotrichum gloeosporioides*, have important roles in oxidative stress response and are critical for virulence (Guo et al., 2011; Sun et al., 2016; Lin et al., 2018). The redox-responsive YAP1 homolog MFAP1 upregulates redox- and virulence-related genes in *Monilinia fructicola* (Yu et al., 2017). In some other species, including *C. heterostrophus* and *Botrytis cinerea* (Temme and Tudzynski, 2009; Montibus et al., 2013) there is only a minor contribution, or fAP1 is entirely dispensable for virulence, despite its role in oxidative stress response (Temme and Tudzynski, 2009). It is not uncommon that virulence phenotypes of the deletion mutants depend on the conditions under which they are assayed; in *C. heterostrophus*, for example, a *Chap1* deletion mutant caused lesions on maize of similar size to the WT when tested by droplet inoculation (Lev et al., 2005), while twofold smaller lesions, on average, were obtained with the same null allele when tested by spray inoculation, on a different maize host (Zhang et al., 2013). Similar to plant pathogens, in the human pathogen *Talaromyces marneffei* yapA is essential for growth, conidiation, pigmentation, response to the oxidative and nitrosative stress. Furthermore, *T. marneffei* yapA is required for survival within human macrophages THP-1 (Dankai et al., 2016). In contrast, in *Aspergillus fumigatus*, which is exposed to ROS originating from neutrophils during infection of the mammalian host, AfYAP1 is dispensable for survival in the presence of polymorphonuclear leukocytes and for virulence in a murine model (Lessing et al., 2007; Qiao et al., 2008). On the other hand, recently, it was found that deletion of the cytosolic peroxiredoxins Prx1 (Cys-based peroxidases) in *A. fumigatus*, whose expression is regulated by AfYap1 and SakA MAP kinase of the HOG pathway, cause a reduction of virulence in a neutropenic murine infection model (Rocha et al., 2018). fAP1, like YAP1, is activated also by diverse stimuli in addition to ROS. In *A. alternata*, fAP1 with Hog1 MAP kinase and Skn7 response regulator mediate resistance by MFS transporter, AaMFS19, to oxidants and different fungicides and chemicals like, clotrimazole, fludioxonil, copper, eosin Y, rose Bengal (RB), hematoporphyrin (HP), methylene blue (MB), and cercosporin (Chen et al., 2017). Recently, another MFS transporter, AaMFS54, has been discovered to increase the resistance of *A. alternata* to fungicides and xenobiotics (Lin et al., 2018). Furthermore, CgAP1 is involved in the regulation of cell wall integrity in *C. gloeosporioides*, which may lead to more resistance to environmental chemical stresses (Li et al., 2017).

#### Transcriptional Targets of YAP1/fAP1 Depend on the Activating Stimulus

Similar to Yap1, *C. heterostrophus* ChAP1 triggers expression of antioxidant genes in response to hydrogen peroxide (Lev et al., 2005). In response to plant phenolics, however, nuclear ChAP1 does not elicit similar changes in gene expression (Shanmugam et al., 2010). Furthermore, phenolic compounds appear to trigger nuclear localization of ChAP1 irrespectively of the oxidative status of the cell: experiments with a genetically encoded reporter of intracellular redox state in *C. heterostrophus* showed a more reduced, rather than oxidized, state upon exposure to phenolics (Ronen et al., 2013). Data collected for YAP1 activation by green tea extract indirectly suggest a similar response in *S. cerevisiae*: nuclear targeting of Yap1 without induction of the downstream antioxidant genes (Takatsume et al., 2005). Additional partners of Yap1/fAP1 could provide the specificity needed for the same transcription factor to activate expression of different sets of genes, as discussed for yeast, above, and in the Section “Signaling Network.” Our working hypothesis is that the link between xenobiotic and oxidative stress response mechanisms (though not necessarily the molecular details) is evolutionarily conserved. Some insight might then be obtained by comparison with the KEAP/Nrf2 pathway.

### Convergent Biochemical Principles – Mammalian Keap1-Nrf2

In mammalian cells the bZip transcription factor Nrf2 and its interacting cysteine-rich protein Keap1 form a core of the pathway activated in response to oxidative and chemical stress (Ma, 2013; Yamamoto et al., 2018). Nrf2 regulates the expression of around 1055 genes (Malhotra et al., 2010) via binding to ARE (Antioxidant Response Element, 5′-GTGACNNNGC-3′) in their promoters (Rushmore et al., 1991). Nrf2 targets include numerous cytoprotective genes which mediate response to oxidative stress, and detoxification of xenobiotics. Keap1-Nrf2 system is extraordinarily flexible: it can be activated by stimuli as diverse as diesel exhaust, food polyphenols, heavy metals and hydrogen peroxide. Furthermore, Nrf2 responds to the intracellular stimuli, namely ER stress (reviewed in Cullinan and Diehl, 2006). The Nrf2 pathway links redox responses and intermediary metabolism (Hayes and Dinkova-Kostova, 2014). Indeed, a recent high-throughput study identified a compound that is not an obvious electrophile, but activates the Keap1-Nrf2 pathway via the reactive endogenous metabolite methylglyoxal. The plasticity of Keap1-Nrf2 system allows modulation of Nrf2 activity via a variety of inputs, including transcriptional regulation, phosphorylation, ubiquitination, acetylation, subcellular compartmentalization, and direct interaction with thiol-reactive xenobiotics, reviewed by Ma (2013) and Silva-Islas and Maldonado (2018).

In the absence of stress, Nrf2 is sequestered in the cytosol in a complex with Keap1, which serves as a substrate adaptor protein for Cullin3-dependent E3 ubiquitin ligase. Keap1 mediates ubiquitination and degradation of Nrf2 by the proteasome system, keeping the abundance of Nrf2 low under non-stressful conditions. Some of the inducers, such as toxic metals As^3+^, Cd^2+^, and Cr^6+^, cause dissociation of Nrf2 from Keap1, and thus stabilization of Nrf2, as shown for example by He et al. (2006) for induction by arsenic (see Figure 1A). Activating compounds react with thiol groups of cysteines of Keap1 and/or Nrf2, depending on the compound, and alter the structure of Keap1-Nrf2-Cul3 complex, reviewed by Dinkova-Kostova et al. (2017). Human Keap1 contains 27 cysteines, all reactive to various degree. Involvement of individual cysteines in Keap1-Nrf2 activation is stimulus-dependent (summarized in Holland et al., 2008). Multiple cysteines of Keap1 and 6–7 cysteines of Nrf2 ensure plasticity of this signaling mechanism, culminating in Nrf2 activation. Inducers such as tert-butylhydroquinone (tBHQ) bind Keap1 cysteines and prevent Nrf2 ubiquitination, but do not disrupt Keap1-Nrf2 complexes. Stabilization of Keap1-bound Nrf2 decreases the amount of free Keap1, and newly synthesized Nrf2 translocates to the nuclei (reviewed in Ma and He, 2012). Nuclear Nrf2 forms heterodimers with small Maf proteins via its bZIP domain and binds to the ARE of target genes to trigger their expression (reviewed in Li et al., 2006; He and Ma, 2009; Ma, 2013; Silva-Islas and Maldonado, 2018). Nrf2 is positioned within a complex regulatory network of interacting proteins which governs activation of its target ARE-containing genes. Some of the Nrf2-regulated genes are activated independently of the stress stimulus and the cellular context (dubbed “default Nrf2 program”), while others are specific to the cell type and the nature of the inducing agent (reviewed in Tonelli et al., 2017).

Functionally, activation of Nrf2 by the two different types of stress, oxidative and chemical, closely resembles activation of YAP1, as noticed previously (Li et al., 2006; Shanmugam et al., 2010; Ouyang et al., 2011). A strong parallel was recently drawn by Fuse and Kobayashi (2017). In addition to the KEAP1-dependent mechanism summarized above (Figure 1), there is evidence for KEAP-1 independent nuclear targeting of Nrf2. Nrf2 has an N-terminal domain, Neh2, that binds KEAP1, transactivation (TA) domains Neh4 and Neh5, followed by the CNC (Cap’n Collar transcription factor motif), a basic region within which there is a NLS, and a C-terminal leucine zipper domain, within which there is a NES. Relevant to the comparison of the metazoan Keap1-Nrf2 signaling system to fungal YAP1, the transactivation domain Neh5 of Nrf2 harbors an additional functional NES with an imbedded reactive cysteine (Cys183 in human Nrf2) (Li et al., 2006). This cysteine was shown to be redox-sensitive and sufficient to prevent nuclear export of Nrf2 GFP-NES_TA_ upon exposure to thiol-reactive chemicals, including tBHQ and the natural isothiocyanate sulforaphane (Li et al., 2006). This Keap1-independent nuclear targeting of Nrf2_TA_ closely resembles the mechanism of YAP1 activation in that no additional partner would be required to promote nuclear retention in response to stress.

**FIGURE 2:**
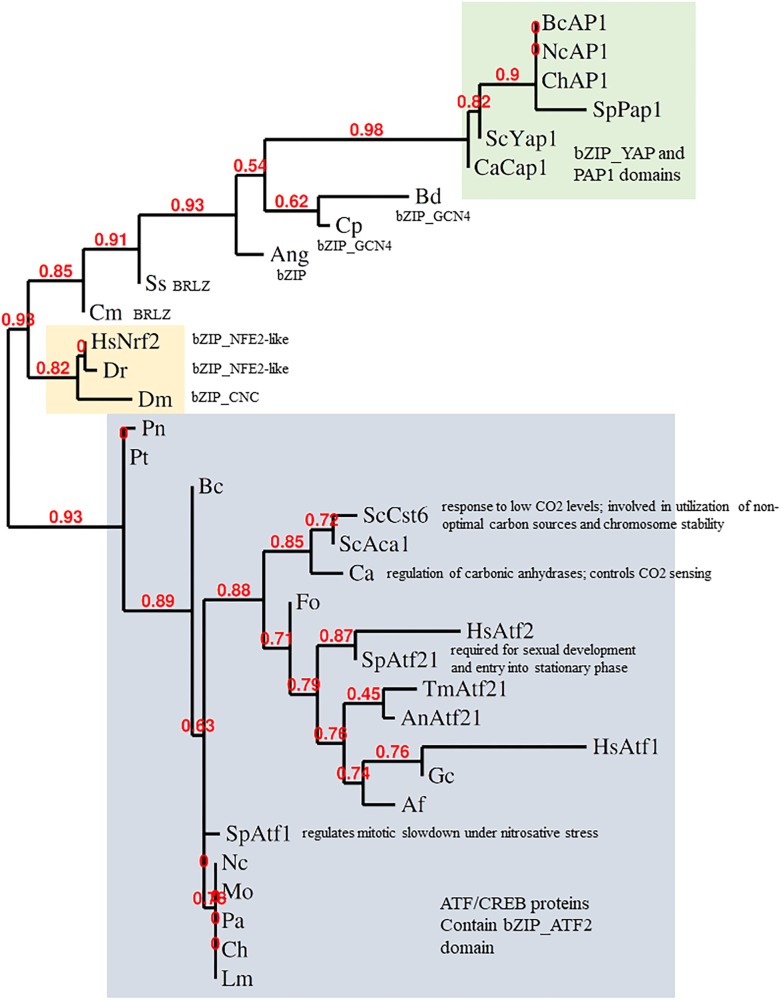
Phylogeny of stress-responsive transcription factors. Analysis of the bZIP domains of metazoan Nrf2 and fungal AP-1 was done by alignment with MUSCLE, curation by Gblocks, phylogeny by PhyML 3.0 and tree rendering by TreeDyn at www.phylogeny.fr (Dereeper et al., 2008). fAP1 and YAP1 are shaded green; Atf1-like gray; Nrf2 orange. BcAP1 XP_024552578.1 Bap1 (*Botrytis cinerea*); NcAP1 CAB91681.2 related to AP-1-like transcription factor (*Neurospora crassa*); ChAP1 AAS64313.1 Chap1 (*Bipolaris maydis*); SpPap1 NP_593662.1 transcription factor Pap1/Caf3 (*Schizosaccharomyces pombe*); ScYap1 NP_013707.1 DNA-binding transcription factor YAP1 (*Saccharomyces cerevisiae*); CaCap1 AAD00802.1 Cap1 (*Candida albicans*); Bd XP_006677485.1 hypothetical protein (*Batrachochytrium dendrobatidis*); Cp CCE42108.1 hypothetical protein (*Candida parapsilosis*); Ang XP_001401817.1 hypothetical protein (*Aspergillus niger*); Ss ERT02908.1 hypothetical protein (*Sporothrix schenckii*); Cm EGX91616.1 basic-leucine zipper (bZIP) transcription factor (*Cordyceps militaris*); Dm NP_001262865.1 cap-n-collar, isoform P (*Drosophila melanogaster*); Dr XP_005172568.1 nuclear factor erythroid 2-related factor 2 (*Danio rerio*); HsNrf2 AAB32188.1 Nrf2 (*Homo sapiens*); Pt XP_001932327.1 conserved hypothetical protein (*Pyrenophora tritici-repentis*); Pn putative uncharacterized protein (*Phaeosphaeria nodorum*); ScCst6 NP_012228.1 Cst6p (*Saccharomyces cerevisiae*); ScAca1 NP_010964.3 Aca1p (*Saccharomyces cerevisiae*); Ca XP_719061.1 Rca1p (*Candida albicans*); Fo EXA38281.1 hypothetical protein (*Fusarium oxysporum*); HsAtf2 EAX11109.1 activating transcription factor 2 (*Homo sapiens*); HsAtf1 XP_011536690.1 cyclic AMP-dependent transcription factor ATF-1 (*Homo sapiens*); SpAtf21 NP_595707.1 Atf-CREB family transcription factor Atf21 (*Schizosaccharomyces pombe*); AnAtf21 CBF71585.1 TPA: bZIP transcription factor (Atf21), putative (*Aspergillus nidulans*); TmAtf21 XP_002145081.1 bZIP transcription factor (Atf21), putative (*Talaromyces marneffei*); SpAtf1 BAA09841.1 atf1 (*Schizosaccharomyces pombe*); Af XP_753103.1 bZIP transcription factor (BACH2), putative (*Aspergillus fumigatus* Af293); Gc EFX04316.1 bzip transcription factor (*Grosmannia clavigera*); Mo XP_003715195.1 BZIP transcription factor (*Magnaporthe oryzae*); Ch XP_014080207.1 hypothetical protein (*Bipolaris maydis*); An EAA62093.1 hypothetical protein (*Aspergillus nidulans*) AnAP1; Nc XP_011394512.1 ascospore lethal-1, variant (*Neurospora crassa*); Lm XP_003844841.1 hypothetical protein (*Leptosphaeria maculans*); Pa XP_001910874.1 uncharacterized protein (*Podospora anserina*).

The Keap1-Nrf2 system is conserved from zebrafish to humans. Indeed, Nrf2 and YAP1 are both bZIP transcription factors. The bZIP class is large and diverse, though, so one must ask how far the comparison can be taken at the sequence and functional levels. It was proposed from phylogenies of the conserved bZIP domain that fungal genomes encode orthologs of Nrf2 (Gacesa et al., 2015, 2016). Some fungal bZIP TFs with (distant) similarity to Nrf2 are shown in Figure 2. The fungal bZIP transcription factors in these phylogenies are, actually, closer to the ATF-CREB family than to YAP1. Clear sequence similarity between Nrf2 and fAP1 is, however, limited to the bZIP domain, suggesting that the similarity in the stress sensing and activation mechanisms between Nrf2 and fAP1 is functional, an instance of the convergent evolution of related bZIP transcription factors, rather than conservation at the sequence level. Interestingly, phylogenetic analysis of Nrf2 and its homologs in fungi using bZIP domain sequence demonstrates that transcription factors defined as Atf21 (contain ATF/CREB type bZIP domain) are the closest to Nrf2 in fungi. YAP1 and fAP1 are classified into a separate branch, all members of which contain cysteine-rich PAP1 domain (Figure 2).

Nrf2 sequences clearly group separately from both fungal ATF1 and YAP1 sequences. In response to oxidants, ATF1 acts downstream of Hog1 in *S. pombe*. A few filamentous fungal ATF1 orthologs have been functionally characterized. BcAtf1 of the gray mold pathogen *B. cinerea* is mainly involved in development and secondary metabolism, and is not required for tolerance toward oxidant and osmotic stress (Temme et al., 2012). Thus, mechanistically speaking, YAP1 shares much similarity with Keap1-Nrf2, but not at the sequence level. Conservation of the bZIP domain in the context of overall sequence divergence could imply that it is important to preserve DNA binding capacity and specificity, while the activating stimulus and/or interacting partners might change under the evolutionary pressure. It seems the DNA binding/dimerisation domains are more conserved than the rest of the protein. The C-terminal zinc finger-containing domain of cryptococcal Crz1, for example, is highly similar to other fungal CRZ transcription factors, while the N-terminal domain is unique to *C. neoformans* (Lev et al., 2012). If the fungal homologs of Nrf2 belong to the Atf2 class, which has generally minor roles in stress response and major roles in development, YAP1/fAP1 may be the functional cognate of Nrf2 despite having less sequence similarity. In metazoans, Nrf2 is localized to the nucleus following release from the grip of the KEAP1 complex that holds it a low level under non-stressed conditions. In fungi, in contrast, the oxidant-triggered conformational change of YAP1/fAP1 allows nuclear retention (Figure 1). How then, do chemical signals activate fAP1? Oxidized and reduced YAP1 have different electrophoretic mobility when resolved under non-reducing conditions (Delaunay et al., 2002) and such assays could demonstrate disulfide formation in fAP1 upon exposure to small-molecule inducers. A complementary approach could be to mutagenize the critical n-CRD cysteines, preventing intramolecular disulfide formation while permitting a (hypothetical) direct reaction of chemical inducers with the c-CRD cysteine residue(s) neighboring the predicted NES.

**FIGURE 3:**
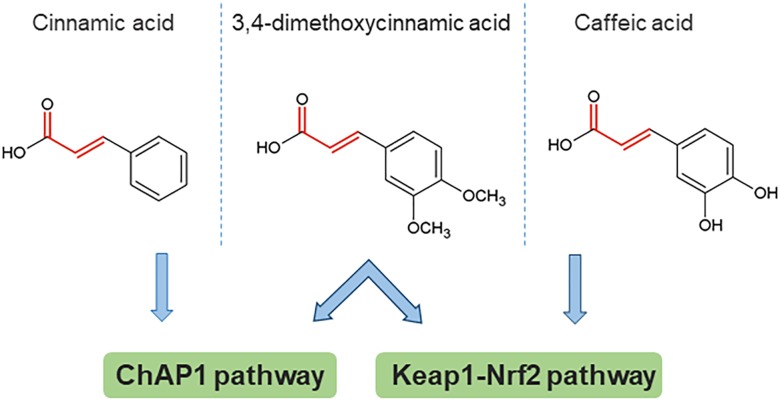
An example of structural determinants for proposed activation of stress response pathways by direct reaction of cinnamic acid derivatives with target cysteines. The Michael acceptor bond structure is emphasized in red. 3,4-dimethoxycinnamic acid is an active inducer common to all three studies (note that the compound abbreviated DMCA in the caffeic acid study is identical to the structure of 3,4-dimethoxycinnamic acid). Based on data from Dinkova-Kostova et al. (2001); Shalaby et al. (2012), Sirota et al. (2015).

Some insight toward the mechanism by which phenolic compounds activate fAP1 can be obtained from studies on Keap1-Nrf2, where reactive cysteine residues act as sensors for both oxidants and chemical inducers with electrophilic properties. The main property common to inducers of phase 2 enzymes is reactivity with sulfhydryl groups. A structure-activity study for the recognition of aromatic compounds by the cellular sensor [likely, but not necessarily, KEAP1 (Dinkova-Kostova et al., 2001]; this was proven by mass spectrometry for a different set of inducers (Dinkova-Kostova et al., 2002) showed that the inducer strength of Michael reaction acceptors was increased by the presence of ortho- (but not other) hydroxyl substituent(s) on the aromatic ring(s). Could these properties be universal, and generalized to fAP1? Apparently not, because cinnamic acid without an ortho OH substituent was the most active compound in nuclear retention of ChAP1, however, this compound was not active in inducing the response of a phase 2 reporter gene (Dinkova-Kostova et al., 2001). Another study with cancer cell lines found that caffeic acid indeed activates the Nrf2 pathway. Caffeic acid has both reducing and electrophilic properties. An analog lacking the Michael acceptor electrophile structure was ineffective in inducing Nrf2 nuclear accumulation and expression of some phase II enzyme-encoding genes. This result points to the importance of the electrophile property, although in the same study there was also evidence for an indirect mechanism in which production of H_2_O_2_ outside the cell contributes (Sirota et al., 2015). Initial observations indicate that generation of ROS by exposure to phenolics is not the main route for ChAP1 activation of *C. heterostrophus*, because phenolics brought the cytoplasm to a more reduced rather than oxidized state (Ronen et al., 2013); these experiments, however, have been done only at one time point and would need to be extended. A schematic structure-activity comparison is shown in Figure 3, from which it can be inferred that there are some common principles that apply to phase II enzyme induction in mammalian cells and fAP1 activation in a fungal plant pathogen. Strikingly, 3,4-dimethoxycinnamic acid activates both these evolutionarily distant stress-responsive systems. The structural determinants for activation are not identical, though, in the different systems.

## Signaling Network

Specificity of fAP1 to the activating stimulus could be determined at different levels: at the promoters of target genes (different transcription factor complexes), or by integration of the outputs of several regulatory hierarchies (for example, MAPK and fAP1 pathways). In yeast, YAP1 does not act alone in response to oxidants. As summarized above, the intramolecular disulfide bond(s) are formed in stepwise and interacting fashion, making the glutathione peroxidase ORP1/Gpx3 an indispensable partner. A glutathione peroxidase of rice blast, MoHyr1, is needed for full virulence; *hyr1* mutants permit stronger accumulation of ROS in the infection court and are defective in ROS-regulated gene expression (Huang et al., 2011). In yeast, the YAP1 binding protein Ybp1 acts, apparently as a scaffold, to organize ORP1/Gpx3 into a ternary complex that promotes condensation of the Orp1 sulfenylated cysteine with one of the six Yap1 cysteines, rather than allowing intramolecular disulfide formation in Orp1 (Bersweiler et al., 2017). Filamentous fungal orthologs of Ybp1 have not been studied yet. Veal et al. (2003) identified a *C. albicans* ortholog, while our initial BLASTP searches of the *C. heterostrophus* database did not give any hits. Ybp1 could have emerged within the Saccharomycotina lineage, or alternatively, lack sufficient sequence homology for easy identification in other ascomycete lineages. It would be worth pursuing yeast two-hybrid screens with filamentous fungal Hyr3/Gpx3 or fAP1 to search for a Ybp1 ortholog, a divergent protein providing a similar scaffolding function, or other novel fAP1 partners. Gpx2/Opr1/Hyr3 and Ybp1 act closely in the activation reactions of yeast YAP1. Widening the circle, the two-component system response regulator Skn7, which is itself a transcription factor, acts together with YAP1 at some oxidant-responsive promoters. ChAP1 and Skn7 co-regulate a subset of antioxidant genes (Shalaby et al., 2014). As discussed above, the coactivator Mediator participates in transcriptional activation of some yeast YAP1 targets, in a gene-specific manner (Gulshan et al., 2005; Lee et al., 2013). ChIP characterization of the transcription factor and coactivator complexes at different fAP1 targets could tell whether Mediator provides signal specificity at the promoters of target genes in filamentous species as well. In particular, oxidant-activated YAP1 may recruit Mediator subunit Rox3 (Gulshan et al., 2005), while nuclear YAP1 imported by a different mechanism might not. The way in which YAP1 is targeted to the nucleus, therefore, would determine the set of target genes. Aside from these direct interaction partners, in yeasts other cellular signaling pathways interact with the YAP1 pathway. In fission yeast, for example, antioxidant genes are co-regulated by PAP1 and by the stress-activated (Hog1) MAPK pathway acting upstream of Atf1 (Toone et al., 1998).

### MAPK Pathways Sensing Different Stresses

MAPK pathways are central to fungal development and pathogenicity (Bahn et al., 2007; Zhao et al., 2007; Hamel et al., 2012). The first fAP1 to be characterized in detail, ChAP1 of the maize pathogen *C. heterostrophus*, was actually discovered while searching for possible targets of the pathogenicity-associated MAP kinase in the genome databases (Lev et al., 2005). Filamentous pathogens of plants require the MAPK Pmk1 (Xu and Hamer, 1996) for disease related development, and it was later shown that the three fungal MAPKs have diverse and sometimes essential virulence roles in different pathogen species. The rationale, originally, was that mammalian AP1 hetero/homodimers of MAPK substrate TF’s suggested that fungal AP1’s might be MAPK substrates too. Nevertheless, after more than a decade of study of fAP1s there is still no clear evidence implicating YAP1 or its filamentous fungal orthologs as substrates for phosphorylation by MAPKs, and the link between MAPK and YAP1 pathways might, rather, be indirect. In the *S. pombe* model, Sty1 (ortholog of Hog1/P338) was placed upstream of PAP1 (and the Atf1 ortholog) yet there as well, evidence is lacking that PAP1 is a Sty1 kinase substrate, rather it was proposed that Sty1 (activated by stress) could interact with PAP1 and facilitate nuclear import (Toone et al., 1998).

ChAP1 contains numerous predicted phosphorylation sites, some with high (>0.9) scores according to NetPhos 3.1^1^ (Blom et al., 2004). In initial phosphoproteomic data, however, we have detected only two phosphorylations of ChAP1, on either serine of the sequence AGAKTQR**S**G**S**LNG located near the central part of the polypeptide (Tamar Ziv, Smoler Protein Structure Center, Technion, 2017, unpubl.). Both are predicted PKA sites, among other kinase predictions. MAPKs are not in the list of NetPhos 3.1 predictions for these two serines, and the highest score prediction (>0.9) is for “unspecified” kinase(s). Looking across the whole ChAP1 molecule there are seven predicted p38 MAPK sites, all just passing the 0.5 score cutoff; interestingly, there are no sites predicted for the other two MAPKs known to be encoded by the *C. heterostrophus* genome. These preliminary observations and predictions would suggest that fAP1 is not a direct phosphorylation target of Hog1, though the phosphoproteome would need to be studied under a wider range of physiological conditions and interaction with the host.

**FIGURE 4:**
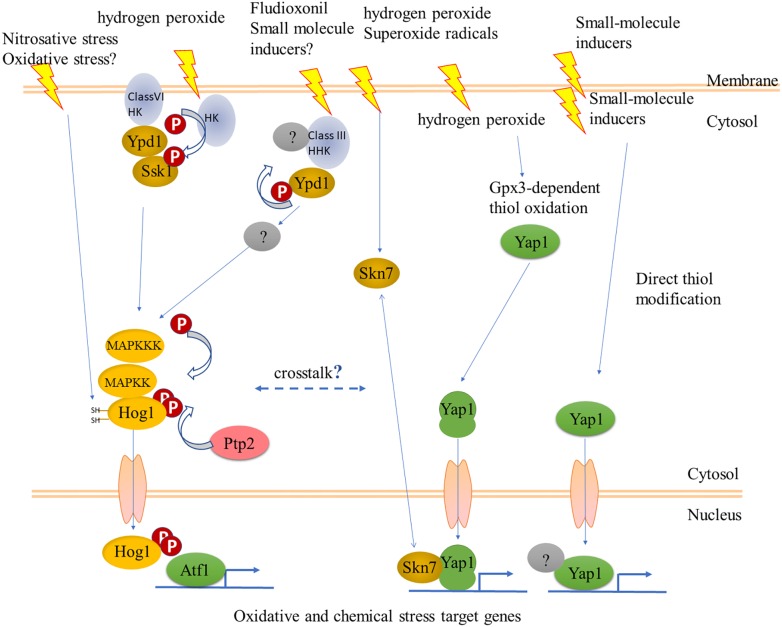
Some possible interactions between fungal cell regulatory hierarchies that could respond to oxidants and chemical stresses. The YAP1 pathway is as outlined in Figure 1. Sensory histidine kinases (HK) signal to the stress-activated MAPK Hog1 via the phosphorelay Ypd1 and response regulator (RR) Ssk1. The mode of action of the fungicide fludioxonil could suggest a mechanism for sensing natural chemical signals. The Class VI HK, Sln1, a transmembrane protein, senses osmotic stress but has not been shown to respond to oxidants, while a different HK has this role in fission yeast. The long arrow to Hog1 in the left side indicates direct activation of Hog1 via reactive cysteines, as shown for nitrosative stress in *Candida albicans* (Herrero-de-Dios et al., 2018); overexpression of the phosphatase Ptp2 was shown to provide resistance to fludioxonil (Bohnert et al., 2018). See text for more details.

Could the Hog1 pathway, nevertheless, be another link between oxidant and chemical stress sensing? Sustained activation of Hog1 routes yeast cells toward programmed/regulated death (RCD) (Vendrell et al., 2011). *C. albicans* cells can adapt to sustained activation of Hog1 by several mechanisms allowing survival (Day et al., 2017). The fungicide fludioxonil, rather than inhibiting a biochemical pathway, acts by hyperactivating the Hog1 pathway, as shown in several filamentous pathogens of plants, and in *Aspergillus nidulans* (Kojima et al., 2004; Lara-Rojas et al., 2011). A link between Hog1 and cell death was delineated in *C. heterostrophus*, where exposure to ferulic acid in millimolar concentrations causes cell death. Ferulic acid also decreased the steady-state phosphorylation levels of Hog1 and Pmk1, while transiently increasing that of the cell-integrity MAPK Mps1. Ferulic acid and fludioxonil had opposite effects on Hog1 phosphorylation, suggesting that as in yeast, reducing sustained activation of Hog1 promotes survival (Shalaby et al., 2016). These *in vitro* experiments have not been extended to infection on the plant. Such rebalancing of information flow through the three MAPK signaling pathways upon exposure to a chemical stress does, however, already raise questions about what happens when chemical and oxidant stresses are combined, when the fungus encounters host defenses. SakA/Hog1 and AtfA/Atf1 cooperate in developmental pathways (Lara-Rojas et al., 2011). More recently it was shown that SakA/Hog1 and NapA/fAP1 also cooperate (Mendoza-Martínez et al., 2017). Thus the *S. pombe* model generalizes to filamentous species. Filamentous fungal genomes encode many more members of two-component (histidine kinase – response regulator) sensory systems than do yeast (Catlett et al., 2003), however, their functions remain largely unknown though recent work is filling this gap (Jacob et al., 2014). One notable exception where a focused function was found is the HK NikA (Hik1 in *M. oryzae*), which is the sensor for fludioxonil (hyper)activation of the Hog1 pathway (Motoyama et al., 2005; Furukawa et al., 2012; Lawry et al., 2016). The mechanism is apparently HK-mediated dephosphorylation of the phosphorelay Ypd1, leading in turn to overactivation of Hog1 (Lawry et al., 2016). Hyperactivation is not what the HKs have evolved for, and one might propose that in normal physiology HK sensors provide an input to integrate chemical and oxidant signals at Hog1 (Figure 4). This hypothesis can be tested by construction of double mutants in fAP1 and individual or combined loss of function in filamentous fungal HKs. The RRs that integrate signals from HKs can also be targets for this strategy (Catlett et al., 2003), keeping in mind though that the phenotypes of RR mutants are more pleiotropic than those of individual HKs. In this context, hybrid HKs like the one responding to fludioxonil might not signal through the known RRs. Compared to studies on protein kinases, dephosphorylation has been (relatively) overlooked, yet together with kinase activity determines the level of activation. Fungal protein phosphatases play critical roles in development (Yatzkan et al., 1998) and have been systematically identified in *Neurospora* (Ghosh et al., 2014; Winkelströter et al., 2015). Most recently, overexpression of MoPTP2, a *M. oryzae* phosphatase that dephosphorylates Hog1, was found to confer resistance to fludioxonil (Bohnert et al., 2018).

In fungal photobiology, a light-stress link was uncovered between light and Hog1/P38 (Vitalini et al., 2007; Esquivel-Naranjo et al., 2016; Yu et al., 2016). The association discussed here between xenobiotic and oxidant sensing is indeed reminiscent of the link between light and osmotic/oxidant sensing. The analogy can be taken still further, as some host-derived compounds act as both signals and stresses; the same is true for light which provides positional and regulatory information, while blue light is also a cue for impending UV stress. Thus the same approaches, the candidate approach as applied to an available *hog1* mutant (Esquivel-Naranjo et al., 2016), and the initially unbiased approach of Yu et al. (2016) could be applied to extend the very incomplete knowledge of the network within which fAP1 acts to link oxidant and chemical stress sensing. In both *Aspergillus* and *Trichoderma*, the upstream signaling elements of the Hog1 pathway were found to be involved, suggesting that the Hog1 pathway is a hub for environmental signals. It will be interesting to see if and how host-derived small molecule signals might also converge onto this hub.

## Outlook

In this note, we have made some connections between findings from different fungal species and from cell biology outside the fungal kingdom, together suggesting that the link between oxidant and chemical stress responses is a functionally conserved one. The extensive knowledge from budding and fission yeasts and from genetic models like *A. nidulans* can suggest how this might work in pathogenic species. From the mechanistic point of view, the site directed mutagenesis approaches used in yeast can be extended to filamentous fungal fAP1. We have discussed the YAP1/fAP1 transcription factors as if there is only one such protein, however, there are eight AP1-like paralogs encoded by the yeast genome, and the roles of fAP1 paralogs in filamentous species need to be addressed in future experiments. Transcriptomic data can now be rapidly accumulated to define the oxidant and chemical stresses regulons in different species, in wild type as compared to signaling mutants. Finally, recent ideas about targeting reactive cysteines for drug design (Long and Aye, 2017) could be applied in search of novel antifungal compounds and strategies.

## Author Contributions

SL originated the comparison between YAP1 and Keap1-Nrf2 systems. HS and SL prepared the diagrams and models. BH coordinated the writing. All authors listed have made a substantial, direct and intellectual contribution to the work, and approved it for publication.

## Conflict of Interest Statement

The authors declare that the research was conducted in the absence of any commercial or financial relationships that could be construed as a potential conflict of interest.

## Footnotes

1 http://www.cbs.dtu.dk/services/NetPhos/

## References

[B1] Alarco A. M. Balan I. Talibi D. Mainville N. Raymond M. (1997). Ap1-mediated multidrug resistance in *Saccharomyces cerevisiae* requires FLR1 encoding a transporter of the major facilitator superfamily. *J. Biol. Chem.* 272 19304–19313. 10.1074/jbc.272.31.19304 9235926

[B2] Alarco A. M. Raymond M. (1999). The bZip transcription factor Cap1p is involved in multidrug resistance and oxidative stress response in *Candida albicans*. *J. Bacteriol.* 181 700–708. 9922230 10.1128/jb.181.3.700-708.1999 PMC93433

[B3] Azevedo D. Tacnet F. Delaunay A. Rodrigues-Pousada C. Toledano M. B. (2003). Two redox centers within Yap1 for H2O2 and thiol-reactive chemicals signaling. *Free Radic. Biol. Med.* 35 889–900. 10.1016/S0891-5849(03)00434-9 14556853

[B4] Bahn Y. S. Xue C. Idnurm A. Rutherford J. C. Heitman J. Cardenas M. E. (2007). Sensing the environment: lessons from fungi. *Nat. Rev. Microbiol.* 5 57–69. 10.1038/nrmicro1578 17170747

[B5] Bersweiler A. D’Autréaux B. Mazon H. Kriznik A. Belli G. Delaunay-Moisan A. (2017). A scaffold protein that chaperones a cysteine-sulfenic acid in H2O2 signaling. *Nat. Chem. Biol.* 13 909–915. 10.1038/nchembio.2412 28628095

[B6] Blom N. Sicheritz-Pontén T. Gupta R. Gammeltoft S. Brunak S. (2004). Prediction of post-translational glycosylation and phosphorylation of proteins from the amino acid sequence. *Proteomics* 4 1633–1649. 10.1002/pmic.200300771 15174133

[B7] Bohnert S. Heck L. Gruber C. Neumann H. Distler U. Tenzer S. (2018). Fungicide resistance towards fludioxonil conferred by overexpression of the phosphatase gene Mo PTP 2 in *Magnaporthe oryzae*. *Mol. Microbiol.* 10.1111/mmi.14179 [Epub ahead of print]. 30537256

[B8] Brown A. J. P. Cowen L. E. di Pietro A. Quinn J. (2017). “Stress adaptation,” in *The Fungal Kingdom*, eds Heitman J. Howlett B. J. Crous P. W. Stukenbrock E. H. James T. Y. Gow N. A. R. (Washington, DC: American Society of Microbiology) 463–485.

[B9] Caetano S. M. Menezes R. Amaral C. Rodrigues-Pousada C. Pimentel C. (2015). Repression of the low affinity iron transporter gene FET4: a novel mechanism against cadmium toxicity orchestrated by Yap1 via Rox1. *J. Biol. Chem.* 290 18584–18595. 10.1074/jbc.M114.600742 26063801 PMC4513117

[B10] Cartwright G. M. Scott B. (2013). Redox regulation of an AP-1-like transcription factor, YapA, in the fungal symbiont *Epichloë festucae*. *Eukaryot. Cell* 12 1335–1348. 10.1128/EC.00129-13 23893078 PMC3811341

[B11] Catlett N. L. Yoder O. C. Turgeon B. G. (2003). Whole-genome analysis of two-component signal transduction genes in fungal pathogens. *Eukaryot. Cell* 2 1151–1161. 10.1128/EC.2.6.1151-1161.2003 14665450 PMC326637

[B12] Chen L.-H. Tsai H.-C. Yu P.-L. Chung K.-R. (2017). A major facilitator superfamily transporter-mediated resistance to oxidative stress and fungicides requires Yap1, Skn7, and MAP kinases in the citrus fungal pathogen *Alternaria alternata*. *PLoS One* 12:e0169103. 10.1371/journal.pone.0169103 28060864 PMC5218470

[B13] Cullinan S. B. Diehl J. A. (2006). Coordination of ER and oxidative stress signaling: the PERK/Nrf2 signaling pathway. *Int. J. Biochem. Cell Biol.* 38 317–332. 10.1016/j.biocel.2005.09.018 16290097

[B14] Dankai W. Pongpom M. Youngchim S. Cooper C. R. Vanittanakom N. (2016). The yapA encodes bZIP transcription factor involved in stress tolerance in pathogenic fungus *Talaromyces marneffei*. *PLoS One* 11:e0163778. 10.1371/journal.pone.0163778 27706212 PMC5051730

[B15] Day A. M. Smith D. A. Ikeh M. A. C. Haider M. Herrero-de-Dios C. M. Brown A. J. P. (2017). Blocking two-component signalling enhances *Candida albicans* virulence and reveals adaptive mechanisms that counteract sustained SAPK activation. *PLoS Pathog.* 13:e1006131. 10.1371/journal.ppat.1006131 28135328 PMC5300278

[B16] Delaunay A. Pflieger D. Barrault M. B. Vinh J. Toledano M. B. (2002). A thiol peroxidase is an H2O2 receptor and redox-transducer in gene activation. *Cell* 111 471–481. 10.1016/S0092-8674(02)01048-6 12437921

[B17] Dereeper A. Guignon V. Blanc G. Audic S. Buffet S. Chevenet F. (2008). Phylogeny.fr: robust phylogenetic analysis for the non-specialist. *Nucleic Acids Res.* 36 W465–W469. 10.1093/nar/gkn180 18424797 PMC2447785

[B18] Dinkova-Kostova A. T. Holtzclaw W. D. Cole R. N. Itoh K. Wakabayashi N. Katoh Y. (2002). Direct evidence that sulfhydryl groups of Keap1 are the sensors regulating induction of phase 2 enzymes that protect against carcinogens and oxidants. *Proc. Natl. Acad. Sci.* 99 11908–11913. 10.1073/pnas.172398899 12193649 PMC129367

[B19] Dinkova-Kostova A. T. Kostov R. V. Canning P. (2017). Keap1, the cysteine-based mammalian intracellular sensor for electrophiles and oxidants. *Arch. Biochem. Biophys.* 617 84–93. 10.1016/j.abb.2016.08.005 27497696 PMC5339396

[B20] Dinkova-Kostova A. T. Massiah M. A. Bozak R. E. Hicks R. J. Talalay P. (2001). Potency of michael reaction acceptors as inducers of enzymes that protect against carcinogenesis depends on their reactivity with sulfhydryl groups. *Proc. Natl. Acad. Sci.* 98 3404–3409. 10.1073/pnas.051632198 11248091 PMC30666

[B21] Esquivel-Naranjo E. U. García-Esquivel M. Medina-Castellanos E. Correa-Pérez V. A. Parra-Arriaga J. L. Landeros-Jaime F. (2016). A *Trichoderma atroviride* stress-activated MAPK pathway integrates stress and light signals. *Mol. Microbiol.* 100 860–876. 10.1111/mmi.13355 26878111

[B22] Furukawa K. Randhawa A. Kaur H. Mondal A. K. Hohmann S. (2012). Fungal fludioxonil sensitivity is diminished by a constitutively active form of the group III histidine kinase. *FEBS Lett.* 586 2417–2422. 10.1016/j.febslet.2012.05.057 22687241

[B23] Fuse Y. Kobayashi M. (2017). Conservation of the Keap1-Nrf2 system: an evolutionary journey through stressful space and time. *Molecules* 22:436. 10.3390/molecules22030436 28282941 PMC6155405

[B24] Gacesa R. Dunlap W. C. Barlow D. J. Laskowski R. A. Long P. F. (2016). Rising levels of atmospheric oxygen and evolution of Nrf2. *Sci. Rep.* 6:27740. 10.1038/srep27740 27297177 PMC4906274

[B25] Gacesa R. Dunlap W. C. Long P. F. (2015). Bioinformatics analyses provide insight into distant homology of the Keap1–Nrf2 pathway. *Free Radic. Biol. Med.* 88 373–380. 10.1016/j.freeradbiomed.2015.06.015 26117326

[B26] Ghosh A. Servin J. A. Park G. Borkovich K. A. (2014). Global analysis of serine/threonine and tyrosine protein phosphatase catalytic subunit genes in *Neurospora crassa* reveals interplay between phosphatases and the p38 mitogen-activated protein kinase. *G3* 4 349–365. 10.1534/g3.113.008813 24347630 PMC3931568

[B27] Gruhlke M. C. H. Schlembach I. Leontiev R. Uebachs A. Gollwitzer P. U. G. Weiss A. (2017). Yap1p, the central regulator of the *S. cerevisiae* oxidative stress response, is activated by allicin, a natural oxidant and defence substance of garlic. *Free Radic. Biol. Med.* 108 793–802. 10.1016/j.freeradbiomed.2017.05.004 28479370

[B28] Gulshan K. Rovinsky S. A. Coleman S. T. Moye-Rowley W. S. (2005). Oxidant-specific folding of Yap1p regulates both transcriptional activation and nuclear localization. *J. Biol. Chem.* 280 40524–40533. 10.1074/jbc.M504716200 16219769

[B29] Guo M. Chen Y. Du Y. Dong Y. Guo W. Zhai S. (2011). The bZIP transcription factor MoAP1 mediates the oxidative stress response and is critical for pathogenicity of the rice blast fungus *magnaporthe oryzae*. *PLoS Pathog.* 7:e1001302. 10.1371/journal.ppat.1001302 21383978 PMC3044703

[B30] Hamel L.-P. Nicole M.-C. Duplessis S. Ellis B. E. (2012). Mitogen-activated protein kinase signaling in plant-interacting fungi: distinct messages from conserved messengers. *Plant Cell* 24 1327–1351. 10.1105/tpc.112.096156 22517321 PMC3398478

[B31] Hayes J. D. Dinkova-Kostova A. T. (2014). The Nrf2 regulatory network provides an interface between redox and intermediary metabolism. *Trends Biochem. Sci.* 39 199–218. 10.1016/j.tibs.2014.02.002 24647116

[B32] He X. Chen M. G. Lin G. X. Ma Q. (2006). Arsenic induces NAD(P)H-quinone oxidoreductase I by disrupting the Nrf2⋅Keap1⋅Cul3 complex and recruiting Nrf2⋅Maf to the antioxidant response element enhancer. *J. Biol. Chem.* 281 23620–23631. 10.1074/jbc.M604120200 16785233

[B33] He X. Ma Q. (2009). NRF2 cysteine residues are critical for oxidant/electrophile-sensing, kelch-like ECH-associated protein-1-dependent ubiquitination-proteasomal degradation, and transcription activation. *Mol. Pharmacol.* 76 1265–1278. 10.1124/mol.109.058453 19786557 PMC2784728

[B34] Herrero-de-Dios C. Day A. M. Tillmann A. T. Kastora S. L. Stead D. Salgado P. S. (2018). Redox regulation, rather than stress-induced phosphorylation, of a Hog1 mitogen-activated protein kinase modulates its nitrosative-stress-specific outputs. *mBio* 9:e2229-17. 10.1128/mBio.02229-17 29588408 PMC5874921

[B35] Holland R. Hawkins A. E. Eggler A. L. Mesecar A. D. Fabris D. Fishbein J. C. (2008). Prospective type 1 and type 2 disulfides of keap1 protein. *Chem. Res. Toxicol.* 21 2051–2060. 10.1021/tx800226m 18729328 PMC4821167

[B36] Huang K. Czymmek K. J. Caplan J. L. Sweigard J. A. Donofrio N. M. (2011). HYR1-mediated detoxification of reactive oxygen species is required for full virulence in the rice blast fungus. *PLoS Pathog.* 7:e1001335. 10.1371/journal.ppat.1001335 21533213 PMC3077360

[B37] Jacob S. Foster A. J. Yemelin A. Thines E. (2014). Histidine kinases mediate differentiation, stress response, and pathogenicity in *Magnaporthe oryzae*. *Microbiologyopen* 3 668–687. 10.1002/mbo3.197 25103193 PMC4234259

[B38] Kojima K. Takano Y. Yoshimi A. Tanaka C. Kikuchi T. Okuno T. (2004). Fungicide activity through activation of a fungal signalling pathway. *Mol. Microbiol.* 53 1785–1796. 10.1111/j.1365-2958.2004.04244.x 15341655

[B39] Lara-Rojas F. Sánchez O. Kawasaki L. Aguirre J. (2011). *Aspergillus nidulans* transcription factor AtfA interacts with the MAPK SakA to regulate general stress responses, development and spore functions. *Mol. Microbiol.* 80 436–454. 10.1111/j.1365-2958.2011.07581.x 21320182 PMC3108070

[B40] Lawry S. M. Tebbets B. Kean I. Stewart D. Hetelle J. Klein B. S. (2016). Fludioxonil induces Drk1, a fungal group III hybrid histidine kinase, to dephosphorylate its downstream target, Ypd1. *Antimicrob. Agents Chemother.* 61:e1414-16. 10.1128/AAC.01414-16 27872062 PMC5278731

[B41] Lee J. Godon C. Lagniel G. Spector D. Garin J. Labarre J. (1999). Yap1 and Skn7 control two specialized oxidative stress response regulons in yeast. *J. Biol. Chem.* 274 16040–16046. 10.1074/JBC.274.23.16040 10347154

[B42] Lee S. K. Chen X. Huang L. Stargell L. A. (2013). The head module of mediator directs activation of preloaded RNAPII in vivo. *Nucleic Acids Res.* 41 10124–10134. 10.1093/nar/gkt796 24005039 PMC3905900

[B43] Lessing F. Kniemeyer O. Wozniok I. Loeffler J. Kurzai O. Haertl A. (2007). The *Aspergillus fumigatus* transcriptional regulator AfYap1 represents the major regulator for defense against reactive oxygen intermediates but is dispensable for pathogenicity in an intranasal mouse infection model. *Eukaryot. Cell* 6 2290–2302. 10.1128/EC.00267-07 17921349 PMC2168236

[B44] Lev S. Desmarini D. Chayakulkeeree M. Sorrell T. C. Djordjevic J. T. (2012). The Crz1/Sp1 transcription factor of *Cryptococcus neoformans* is activated by calcineurin and regulates cell wall integrity. *PLoS One* 7:e51403. 10.1371/journal.pone.0051403 23251520 PMC3520850

[B45] Lev S. Hadar R. Amedeo P. Baker S. E. Yoder O. C. Horwitz B. A. (2005). Activation of an AP1-like transcription factor of the maize pathogen *Cochliobolus heterostrophus* in response to oxidative stress and plant signals. *Eukaryot. Cell* 4 443–454. 10.1128/EC.4.2.443-454.2005 15701806 PMC549334

[B46] Li W. Yu S. W. Kong A. N. T. (2006). Nrf2 possesses a redox-sensitive nuclear exporting signal in the Neh5 transactivation domain. *J. Biol. Chem.* 281 27251–27263. 10.1074/jbc.M602746200 16790425

[B47] Li X. Wu Y. Liu Z. Zhang C. (2017). The function and transcriptome analysis of a bZIP transcription factor CgAP1 in *Colletotrichum gloeosporioides*. *Microbiol. Res.* 197 39–48. 10.1016/j.micres.2017.01.006 28219524

[B48] Lin C.-H. Yang S. L. Chung K.-R. (2009). The YAP1 homolog–mediated oxidative stress tolerance is crucial for pathogenicity of the necrotrophic fungus *Alternaria alternata* in Citrus. *Mol. Plant Microbe Interact.* 22 942–952. 10.1094/MPMI-22-8-0942 19589070

[B49] Lin C.-H. Yang S. L. Chung K.-R. (2011). Cellular responses required for oxidative stress tolerance, colonization, and lesion formation by the necrotrophic fungus *Alternaria alternata* in citrus. *Curr. Microbiol.* 62 807–815. 10.1007/s00284-010-9795-y 20978890

[B50] Lin H.-C. Yu P.-L. Chen L.-H. Tsai H.-C. Chung K.-R. (2018). A major facilitator superfamily transporter regulated by the stress-responsive transcription factor Yap1 Is required for resistance to fungicides, xenobiotics, and oxidants and full virulence in *Alternaria alternata*. *Front. Microbiol.* 9:2229. 10.3389/fmicb.2018.02229 30279684 PMC6153361

[B51] Long M. J. C. Aye Y. (2017). Privileged electrophile sensors: a resource for covalent drug development. *Cell Chem. Biol.* 24 787–800. 10.1016/j.chembiol.2017.05.023 28648380 PMC5528179

[B52] Ma Q. (2013). Role of Nrf2 in oxidative stress and toxicity. *Annu. Rev. Pharmacol. Toxicol.* 53 401–426. 10.1146/annurev-pharmtox-011112-140320 23294312 PMC4680839

[B53] Ma Q. He X. (2012). Molecular basis of electrophilic and oxidative defense: promises and perils of Nrf2. *Pharmacol. Rev.* 64 1055–1081. 10.1124/pr.110.004333 22966037 PMC4648289

[B54] Maeta K. Izawa S. Okazaki S. Kuge S. Inoue Y. (2004). Activity of the Yap1 transcription factor in *Saccharomyces cerevisiae* is modulated by methylglyoxal, a metabolite derived from glycolysis. *Mol. Cell. Biol.* 24 8753–8764. 10.1128/MCB.24.19.8753-8764.2004 15367692 PMC516737

[B55] Malhotra D. Portales-Casamar E. Singh A. Srivastava S. Arenillas D. Happel C. (2010). Global mapping of binding sites for Nrf2 identifies novel targets in cell survival response through chip-seq profiling and network analysis. *Nucleic Acids Res.* 38 5718–5734. 10.1093/nar/gkq212 20460467 PMC2943601

[B56] Mendoza-Martínez A. E. Lara-Rojas F. Sánchez O. Aguirre J. (2017). NapA mediates a redox regulation of the antioxidant response, carbon utilization and development in *Aspergillus nidulans*. *Front. Microbiol.* 8:516. 10.3389/fmicb.2017.00516 28424666 PMC5371717

[B57] Menezes R. A. Amaral C. Batista-Nascimento L. Santos C. Ferreira R. B. Devaux F. (2008). Contribution of Yap1 towards *Saccharomyces cerevisiae* adaptation to arsenic-mediated oxidative stress. *Biochem. J.* 414 301–311. 10.1042/BJ20071537 18439143

[B58] Molina L. Kahmann R. (2007). An *Ustilago maydis* gene involved in H2O2 detoxification is required for virulence. *Plant Cell Online* 19 2293–2309. 10.1105/tpc.107.052332 17616735 PMC1955693

[B59] Montibus M. Ducos C. Bonnin-Verdal M.-N. Bormann J. Ponts N. Richard-Forget F. (2013). The bZIP transcription factor fgap1 mediates oxidative stress response and trichothecene biosynthesis but not virulence in *Fusarium graminearum*. *PLoS One* 8:e83377. 10.1371/journal.pone.0083377 24349499 PMC3861502

[B60] Motoyama T. Kadokura K. Ohira T. Ichiishi A. Fujimura M. Yamaguchi I. (2005). A two-component histidine kinase of the rice blast fungus is involved in osmotic stress response and fungicide action. *Fungal Genet. Biol.* 42 200–212. 10.1016/j.fgb.2004.11.002 15707841

[B61] Mulford K. E. Fassler J. S. (2011). Association of the Skn7 and Yap1 transcription factors in the *Saccharomyces cerevisiae* oxidative stress response. *Eukaryot. Cell* 10 761–769. 10.1128/EC.00328-10 21478431 PMC3127664

[B62] Okazaki S. Tachibana T. Naganuma A. Mano N. Kuge S. (2007). Multistep disulfide bond formation in Yap1 is required for sensing and transduction of H2O2 stress signal. *Mol. Cell* 27 675–688. 10.1016/j.molcel.2007.06.035 17707237

[B63] Ouyang X. Tran Q. T. Goodwin S. Wible R. S. Sutter C. H. Sutter T. R. (2011). Yap1 activation by H2O2 or thiol-reactive chemicals elicits distinct adaptive gene responses. *Free Radic. Biol. Med.* 50 1–13. 10.1016/j.freeradbiomed.2010.10.697 20971184

[B64] Pimentel C. Caetano S. M. Menezes R. Figueira I. Santos C. N. Ferreira R. B. (2014). Yap1 mediates tolerance to cobalt toxicity in the yeast *Saccharomyces cerevisiae*. *Biochim. Biophys. Acta* 1840 1977–1986. 10.1016/j.bbagen.2014.01.032 24486411

[B65] Qiao J. Kontoyiannis D. P. Calderone R. Li D. Ma Y. Wan Z. (2008). Af yap1, encoding a bZip transcriptional factor of *Aspergillus fumigatus*, contributes to oxidative stress response but is not essential to the virulence of this pathogen in mice immunosuppressed by cyclophosphamide and triamcinolone. *Med. Mycol.* 46 773–782. 10.1080/13693780802054215 18608886

[B66] Rangel D. E. N. Finlay R. D. Hallsworth J. E. Dadachova E. Gadd G. M. (2018). Fungal strategies for dealing with environment- and agriculture-induced stresses. *Fungal Biol.* 122 602–612. 10.1016/j.funbio.2018.02.002 29801805

[B67] Rocha M. C. de Godoy K. F. Bannitz-Fernandes R. Fabri J. H. T. M. Barbosa M. M. F. de Castro P. A. (2018). Analyses of the three 1-Cys peroxiredoxins from *Aspergillus fumigatus* reveal that cytosolic Prx1 is central to H2O2 metabolism and virulence. *Sci. Rep.* 8:12314. 10.1038/s41598-018-30108-2 30120327 PMC6098058

[B68] Ronen M. Shalaby S. Horwitz B. A. (2013). Role of the transcription factor ChAP1 in cytoplasmic redox homeostasis: imaging with a genetically encoded sensor in the maize pathogen *Cochliobolus heterostrophus*. *Mol. Plant Pathol.* 14 786–790. 10.1111/mpp.12047 23745603 PMC6638657

[B69] Rushmore T. H. Morton M. R. Pickett C. B. (1991). The antioxidant responsive element: activation by oxidative stress and identification of the DNA consensus sequence required for functional activity. *J. Biol. Chem.* 266 11632–11639. 1646813

[B70] Shalaby S. Horwitz B. A. Larkov O. (2012). Structure-activity relationships delineate how the maize pathogen *Cochliobolus heterostrophus* uses aromatic compounds as signals and metabolites. *Mol. Plant Microbe Interact.* 25 931–940. 10.1094/MPMI 22452657

[B71] Shalaby S. Larkov O. Lamdan N. L. Goldshmidt-Tran O. Horwitz B. A. (2016). Plant phenolic acids induce programmed cell death of a fungal pathogen: MAPK signaling and survival of *Cochliobolus heterostrophus*. *Environ. Microbiol.* 18 4188–4199. 10.1111/1462-2920.13528 27631532

[B72] Shalaby S. Larkov O. Lamdan N. L. Horwitz B. A. (2014). Genetic interaction of the stress response factors ChAP1 and Skn7 in the maize pathogen *Cochliobolus heterostrophus*. *FEMS Microbiol. Lett.* 350 83–89. 10.1111/1574-6968.12314 24164316

[B73] Shanmugam V. Ronen M. Shalaby S. Larkov O. Rachamim Y. Hadar R. (2010). The fungal pathogen *Cochliobolus heterostrophus* responds to maize phenolics: novel small molecule signals in a plant-fungal interaction. *Cell. Microbiol.* 12 1421–1434. 10.1111/j.1462-5822.2010.01479.x 20438575

[B74] Silva-Islas C. A. Maldonado P. D. (2018). Canonical and non-canonical mechanisms of Nrf2 activation. *Pharmacol. Res.* 134 92–99. 10.1016/j.phrs.2018.06.013 29913224

[B75] Sirota R. Gibson D. Kohen R. (2015). The role of the catecholic and the electrophilic moieties of caffeic acid in Nrf2/Keap1 pathway activation in ovarian carcinoma cell lines. *Redox Biol.* 4 48–59. 10.1016/J.REDOX.2014.11.012 25498967 PMC4309848

[B76] Sun Y. Wang Y. Tian C. (2016). bZIP transcription factor CgAP1 is essential for oxidative stress tolerance and full virulence of the poplar anthracnose fungus *Colletotrichum gloeosporioides*. *Fungal Genet. Biol.* 95 58–66. 10.1016/j.fgb.2016.08.006 27544415

[B77] Sundström L. Larsson S. Jönsson L. J. (2010). Identification of *Saccharomyces cerevisiae* genes involved in the resistance to phenolic fermentation inhibitors. *Appl. Biochem. Biotechnol.* 161 106–115. 10.1007/s12010-009-8811-9 19847383

[B78] Takatsume Y. Izawa S. Inoue Y. (2006). Methylglyoxal as a signal initiator for activation of the stress-activated protein kinase cascade in the fission yeast *Schizosaccharomyces pombe*. *J. Biol. Chem.* 281 9086–9092. 10.1074/jbc.M511037200 16464860

[B79] Takatsume Y. Maeta K. Izawa S. Inoue Y. (2005). Enrichment of yeast thioredoxin by green tea extract through activation of Yap1 transcription factor in *Saccharomyces cerevisiae*. *J. Agric. Food Chem.* 53 332–337. 10.1021/jf048818h 15656669

[B80] Takeuchi T. Miyahara K. Hirata D. Miyakawa T. (1997). Mutational analysis of Yap1 protein, an AP-1-like transcriptional activator of *Saccharomyces cerevisiae*. *FEBS Lett.* 416 339–343. 10.1016/S0014-5793(97)01233-7 9373181

[B81] Temme N. Oeser B. Massaroli M. Heller J. Simon A. Collado I. G. (2012). BcAtf1, a global regulator, controls various differentiation processes and phytotoxin production in *Botrytis cinerea*. *Mol. Plant Pathol.* 13 704–718. 10.1111/j.1364-3703.2011.00778.x 22293085 PMC6638710

[B82] Temme N. Tudzynski P. (2009). Does *Botrytis cinerea* Ignore H(2)O(2)-induced oxidative stress during infection? Characterization of botrytis activator protein 1. *Mol. Plant. Microbe. Interact.* 22 987–998. 10.1001/archotol.1992.01880010022008 19589074

[B83] Toledano M. B. Delaunay A. Monceau L. Tacnet F. (2004). Microbial H2O2 sensors as archetypical redox signaling modules. *Trends Biochem. Sci.* 29 351–357. 10.1016/j.tibs.2004.05.005 15236742

[B84] Tonelli C. Chio I. I. C. Tuveson D. A. (2017). Transcriptional regulation by Nrf2. *Antioxid. Redox Signal.* 29 1727–1745. 10.1089/ars.2017.7342 28899199 PMC6208165

[B85] Toone W. M. Kuge S. Samuels M. Morgan B. A. Toda T. Jones N. (1998). Regulation of the fission yeast transcription factor Pap1 by oxidative stress: requirement for the nuclear export factor Crm1 (Exportin) and the stress-activated MAP kinase Sty1/Spc1. *Genes Dev.* 12 1453–1463. 10.1101/gad.12.10.1453 9585505 PMC316839

[B86] Veal E. A. Ross S. J. Malakasi P. Peacock E. Morgan B. A. (2003). Ybp1 Is required for the hydrogen peroxide-induced oxidation of the Yap1 transcription factor. *J. Biol. Chem.* 278 30896–30904. 10.1074/jbc.M303542200 12743123

[B87] Vendrell A. Martánez-Pastor M. González-Novo A. Pascual-Ahuir A. Sinclair D. A. Proft M. (2011). Sir2 histone deacetylase prevents programmed cell death caused by sustained activation of the Hog1 stress-activated protein kinase. *EMBO Rep.* 12 1062–1068. 10.1038/embor.2011.154 21836634 PMC3185340

[B88] Vitalini M. W. de Paula R. M. Goldsmith C. S. Jones C. A. Borkovich K. A. Bell-Pedersen D. (2007). Circadian rhythmicity mediated by temporal regulation of the activity of p38 MAPK. *Proc. Natl. Acad. Sci.* 104 18223–18228. 10.1073/pnas.0704900104 17984065 PMC2084324

[B89] Wemmie J. A. Szczypka M. S. Thiele D. J. Moye-Rowley W. S. (1994). Cadmium tolerance mediated by the yeast AP-1 protein requires the presence of an ATP-binding cassette transporter-encoding gene, YCF1. *J. Biol. Chem.* 269 32592–32597. 10.1007/BF00280413 7798263

[B90] Winkelströter L. K. Dolan S. K. Fernanda dos Reis T. Bom V. L. P. Alves de Castro P. Hagiwara D. (2015). Systematic global analysis of genes encoding protein phosphatases in *Aspergillus fumigatus*. *G3* 5 1525–1539. 10.1534/g3.115.016766 25943523 PMC4502386

[B91] Wood M. J. Andrade E. C. Storz G. (2003). The redox domain of the Yap1p transcription factor contains two disulfide bonds. *Biochemistry* 42 11982–11991. 10.1021/bi035003d 14556629

[B92] Wood M. J. Storz G. Tjandra N. (2004). Structural basis for redox regulation of Yap1 transcription factor localization. *Nature* 430 917–921. 10.1038/nature02790 15318225

[B93] Xu J. R. Hamer J. E. (1996). MAP kinase and cAMP signaling regulate infection structure formation and pathogenic growth in the rice blast fungus *Magnaporthe grisea*. *Genes Dev.* 10 2696–2706. 10.1101/gad.10.21.2696 8946911

[B94] Yamamoto M. Kensler T. W. Motohashi H. (2018). The KEAP1-NRF2 system: a thiol-based sensor-effector apparatus for maintaining redox homeostasis. *Physiol. Rev.* 98 1169–1203. 10.1152/physrev.00023.2017 29717933 PMC9762786

[B95] Yatzkan E. Szöor B. Fehér Z. Dombrádi V. Yarden O. (1998). Protein phosphatase 2A is involved in hyphal growth of *Neurospora crassa*. *Mol. Gen. Genet.* 259 523–531. 10.1007/s004380050844 9790584

[B96] Yu P.-L. Wang C.-L. Chen P.-Y. Lee M.-H. (2017). YAP1 homologue-mediated redox sensing is crucial for a successful infection by *Monilinia fructicola*. *Mol. Plant Pathol.* 18 783–797. 10.1111/mpp.12438 27239957 PMC6638302

[B97] Yu Z. Armant O. Fischer R. (2016). Fungi use the SakA (HogA) pathway for phytochrome-dependent light signalling. *Nat. Microbiol.* 1:16019. 10.1038/nmicrobiol.2016.19 27572639

[B98] Zhang N. NurAinIzzati M. Z. Scher K. Condon B. J. Horwitz B. A. Turgeon B. G. (2013). Iron, oxidative stress, and virulence: roles of iron-sensitive transcription factor Sre1 and the redox sensor ChAp1 in the maize pathogen *Cochliobolus heterostrophus*. *Mol. Plant Microbe Interact.* 26 1473–1485. 10.1094/MPMI-02-13-0055-R 23980626

[B99] Zhao X. Mehrabi R. Xu J.-R. (2007). Mitogen-activated protein kinase pathways and fungal pathogenesis. *Eukaryot. Cell* 6 1701–1714. 10.1128/EC.00216-07 17715363 PMC2043402

